# A Systematic Computational Study on Flavonoids

**DOI:** 10.3390/ijms11052017

**Published:** 2010-05-03

**Authors:** Santiago Aparicio

**Affiliations:** Department of Chemistry, University of Burgos, 09001 Burgos, Spain; E-Mail: sapar@ubu.es; Tel.: +34-947-258-062; Fax: +34-947-258-831

**Keywords:** flavonoids, DFT, hydrogen bonding, AIM, NBO

## Abstract

17 selected flavones derivatives, flavonoids, were analyzed through a systematic B3LYP/6-311++G** computational study with the aim of understanding the molecular factors that determine their structural and energetic properties in gas phase. Flavonoids were selected in a systematic way to infer the effect of the number and relative positions of hydroxyl groups on molecular properties. Different conformers for each flavonoid were analyzed and the strength and topology of the intramolecular hydrogen bonds studied through the computation of the corresponding torsional profiles. Atoms in a Molecule, and Natural Bond Orbital methodology was applied to the analysis of charge distribution along the studied molecules, and the intramolecular hydrogen bonds. Molecular shapes were studied through full geometry optimization, and the position of the catechol ring analyzed through dihedral scans.

## 1. Introduction

Flavonoids are compounds abundant in many fruits, vegetables and plant tissues [1–4], and thus included in the common daily diet of many human beings [5–7]. They have remarkable biological and medical importance [8,9], and they have attracted great attention because of their promising properties in nutrition and pharmacological fields. One of the most remarkable properties of flavonoids rises from their behavior as antioxidants [10] acting through a variety of ways, including direct inhibition of formation or activity of reactive oxygen species and interaction, inhibition, with enzymes [10–12]. Thus, this family of molecules has a remarkable pharmacological importance as therapeutic agents. They have been directly used, or through some of their derivatives, in the treatment of diseases ranging from allergies [13] to microbial [14], viral [15] or bacterial [16] infectious processes, or even for cancer therapies [17]. The study of flavonoids is therefore of great practical and theoretical importance. Nevertheless, the constant investigation of flavonoids has not led to a clear relationship between their molecular structure and properties. Nonetheless, the physiological activity of these molecules has been related to their structure and geometry [18,19]. Moreover, their remarkable antioxidant properties rise from the formation of radicals, because of the hydroxyaromatic groups, and from the extensive charge delocalization through the molecules [20,21]. Thus, the detailed knowledge of their molecular structures, both from energetic and geometric viewpoints, is of great importance to gain a deeper insight into their possible therapeutic applications.

**Figure 1.**
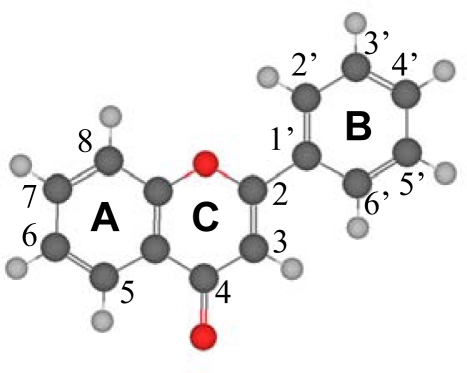
A molecular diagram of Flavone, showing atom numbering and ring names. Structure optimized at B3LYP/6-311++G** theoretical level.

Computational chemistry methods are one of the most powerful tools to achieve advances in this field, and several studies may be found in the literature on this subject [19,21,22–26]. They provide very valuable information at moderate economical costs, prior to time–consuming and expensive experimental or clinical studies, and allow inferring the effects of different molecular features on compounds’ properties. Although some theoretical studies have been published on wide collections of flavonoids [23], most of the literature results are reported only for single compounds without relating them to the whole flavonoid family and shared properties. To the best of my knowledge, no systematic results have been reported considering the effects of hydroxyl groups on the structural and energetic properties of flavonoid compounds. Thus, in this work the results of an extensive study on flavone, Figure 1, and a collection of flavone derivatives, rising from the presence of hydroxyl groups in different molecular positions, are reported. The molecular structure of this family of compounds is characterized by the large charge delocalization spreading along the studied molecules, from A to B rings through the C one because of the C2–C3 double bond. Hence, the main subject of this work is to understand how the presence of hydroxyl groups affects this charge delocalization, and how this is related to geometric and molecular parameters and with molecular energy. The torsional profiles around the C2-C1’ bond, that show the planarity of the molecular moiety and the effect on molecular properties of the rotation of catechol moiety (B-ring), are also studied as a function of the different hydroxyl molecular substituents, and this is also related to the charge delocalization. Intramolecular hydrogen bonding between different positions is also studied. Torsional scans were performed to analyze the strength of these H-bonds and the energetic differences between the different possible conformers. Density functional theory (DFT) was used throughout this work because it provides a compromise between accuracy and computational requirements, considering the large number of compounds and properties studied.

Flavonoids have the same basic structure derived from the parent molecule reported in Figure 1, thus, the 17 compounds studied in this work are reported in Table 1, all of which are direct flavone derivatives with hydroxyl groups placed in the main molecular positions: 5 and 7 in ring A, 3′, 4′ and 5′ in ring B, and 3 in ring C. The number of hydroxyl groups and their positions were varied in a systematic way with the aim of analyzing their effects on molecular properties.

## 2. Computational Methods

DFT calculations were carried out with the Gaussian 03 package [27] according to Density Functional Theory, using the Becke gradient corrected exchange functional [28] and Lee-Yang-Parr correlation functional [29] with three parameters (B3LYP) [30] method. 6-311++G** basis set was used throughout this work. Atomic charges were calculated to fit the electrostatic potential [31] according to the Merz-Singh-Kollman (MK) [32] scheme. Torsional barriers, dihedral scans, were calculated through relaxed scanning of potential energy surfaces at 10° intervals. In this scanning procedure, for each change of the corresponding torsional angle, the structure is fully optimized for the remaining degrees of freedom. Vibrational frequencies were calculated and analyzed to confirm the presence of true minima for the full geometry optimizations by the absence of imaginary values. To more deeply analyze the topological and energetic characteristics of the intramolecular hydrogen bonding and molecular features, Atoms in Molecules (AIM) [33] and Natural Bond Orbital (NBO) [34] studies were also performed. All the calculations were done for the optimized structures in gas phase. AIM calculations were performed using AIM2OOO Software [35].

## 3. Results and Discussion

### 3.1. Structural Features

**Figure 2.**
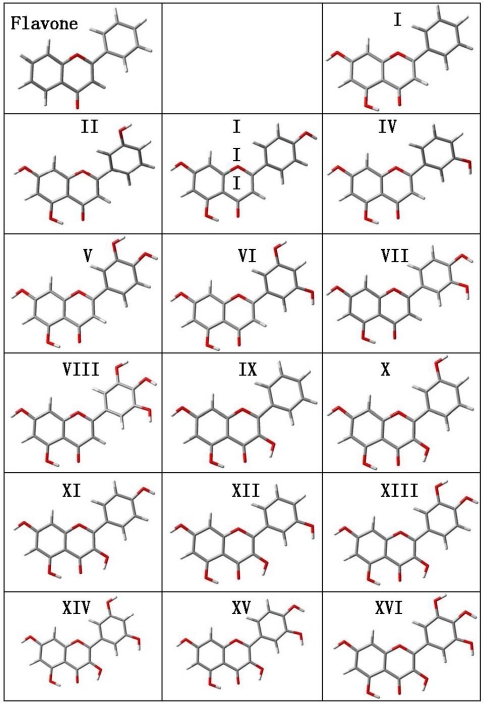
Optimized structures the of studied flavonoids calculated in gas phase at the B3LYP//6-311++G** theoretical level. Atom color code: oxygen (red), carbon (gray) and hydrogen (white).

Structural optimizations were carried out for flavone and the 16 derived flavonoids with the calculated structures reported in Figure 2 and the main molecular parameters in Table 2. Several questions rise from the reported gas phase results. The first one is the planarity of the studied molecules. Reported results show that the dihedral angle between the catechol ring (B ring in Figure 1) and the plane formed by A and C rings is around 20° for flavone and those flavonoids without an hydroxyl group in position 3 of C ring (molecules I to VIII), whereas for those molecules with an hydroxyl group in potion 3, molecules IX to XVI, the dihedral angle is 0° and the three rings, A, B and C, are coplanar. This may be justified considering that the presence of an hydroxyl group in position 3 leads to the development of an intramolecular hydrogen bonding with the hydrogen in position 6′ of B ring. The development of an effective interaction between these positions forces the coplanarity of both rings, and thus, all flavonoids with an hydroxyl group in position 3 on ring C should be planar. Moreover, the π-delocalization from the C ring toward the catechol B-ring will be more effective with this planar structure forced by the 3-OH group. These results are in agreement with the previous findings of van Acker *et al*. [19,36] using the HF/STO-3G approach, whereas other literature results obtained at low theoretical levels (HF/6-31G(d) [37,38] or AM1 [39,40]) report non-planarity for flavonoids with 3-OH groups such as quercetin. These low level theoretical calculations underestimate the effect rising from the rings coplanarity, such as the π-delocalization [37], and thus planar structures obtained in this work should be more reliable. Available X-ray crystal structures lead to molecules almost planar, as is the case for quercetin, for which a 5° experimental angle between cathecol and AC rings is reported [41]. Although packing effects rise in solid phases that are not present in gas phase calculations such as those reported in this work, these crystal results show that even in solid phases, close to planar structures are obtained, thus reinforcing our gas phase conclusions.

**Figure 3.**
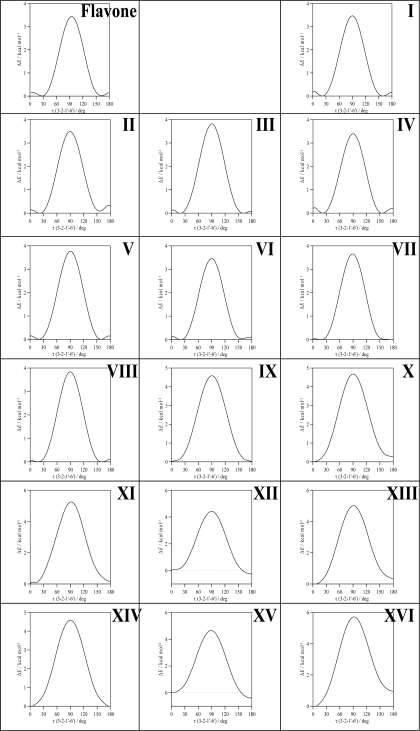
Relaxed potential energy scans of studied flavonoids calculated in gas phase at B3LYP//6-311++G** theoretical level. Scanned dihedral (3-2-1’-6’) with atom numbering as in Figure 1. Δ*E* is the energy relative to the conformer with lower energy for each scan.

To clarify the question of molecular planarity, relaxed potential energy scans were carried out for the (3-2-1’-6’), Figure 1, dihedral angle, for flavone and the 16 studied flavonoids in which the dihedral angle was changed in 10° steps and the remaining molecular parameters were optimized for each fixed dihedral angle. The results are reported in Figure 3 for the whole torsional profiles, and in Table 3, the maxima of the torsional barriers are reported.

All the molecules show a remarkable torsional barrier with maxima for a dihedral angle of 90°, as is to be expected, because the conjugation stabilizing effect for this angle is null. The torsional barriers are different for flavone and molecules I–VIII to those of molecules IX–XVI. For flavone and molecules I–VIII, minima appear at dihedrals around 20°, as we have reported in the previous paragraph, with small maxima for 0° and 180°, whereas for molecules IX–XVI minima are clearly defined for 0° dihedrals. Moreover, the maxima of the torsional barrier for flavone and I–VIII molecules are in the 3.4–3.8 kcal mol^−1^ range, whereas, for the molecules IX–XVI, they are in the 4.4–5.7 kcal mol^−1^ range, Table 2. These facts show that two factors contribute to the structure of the studied molecules: *i*) the conjugation between rings and *ii*) the hydrogen bonding between 3-OH and 6′-H. The first factor is the only one present for flavone and molecules I–VIII, and thus, because of the almost null energetic barrier ongoing from 0° dihedral to 20° dihedral (∼0.2 kcal mol^−1^), the 20° dihedral structure should be preferred because in this case the repulsions between hydrogens in 3 and 6′ are minimized, and at the same time the conjugation between rings is maintained. For molecules IX–XVI, the presence of the hydrogen bonding between 3-OH and 6′-H leads to an additional contribution that increases the rotational barrier, and thus the hydrogen bonding through this position may be confirmed and it should be the responsibility of the planarity of molecules IX–XVI. The additional energy contribution rising from the 3-OH/6’-H hydrogen bonding may be quantified as the difference of the torsional barrier maxima reported in Table 3, that is to say, comparing molecule II with molecule X, molecule III with molecule XI and so on; then, this contribution is around 1 kcal mol^−1^, except for compound XVI (myricetin) for which it is 1.87 kcal mol^−1^. The remaining parameters reported in Table 2 show very weak variations for the studied compounds. 7-OH group interatomic distance is unaffected by the changes in the molecular structure studied in this work. 3-OH group is not affected by the presence of OH groups in the cathecol ring, and, thus, its interaction with the keto group in C-ring and the 6′-H in catechol ring, does not change with the presence of hydroxyl groups in this B-ring. Likewise, hydroxyl groups in the 3′, 4′ and 5′ positions on B ring does not change remarkably with the presence/absence of neighboring hydroxyl groups. Hence, the interaction between C and B rings is done through conjugation, for molecules without 3-OH group, and through this effect and the interaction between 3-OH and 6′-H sites, for molecules with a 3-OH group.

In Table 2, gas phase dipole moments are also reported, flavonoids without 3-OH groups are more polar (moments in the 4–5 D range) than those with 3-OH groups (moments in the 2–3 D range). The knowledge of flavonoids dipole moment is important to understand their biological activity because the interaction of these molecules with biomolecules is developed through hydrogen bonding of the hydroxyl sites and through van der Waals forces [42]. Nonetheless, dipole moment is strongly dependent on very weak structural changes, for example molecule XVI (myricetin) has a dipole moment of 1.50 D for the structure reported in Figure 2, in this molecule, hydroxyl groups in the B ring are clockwise oriented, then if these groups are counter clockwise oriented (the energy difference between both structures is only −0.86 kcal mol^−1^) the dipole moment increases to 5.14 D This is in agreement with literature results at a lower theoretical level [22]. As a rule, flavonoid conformations with hydroxyl groups counter clockwise oriented in the B ring show a remarkably larger dipole moment than those clockwise oriented, and the energy difference between both orientations is very low, but favorable to clockwise orientations. Moreover, the orientation of the 7-OH group leads also to important variations of dipole moment. For molecule XVI (myricetin), the counter clockwise oriented 7-OH group leads to 1.50 D whereas clockwise oriented leads to 5.10 D. Considering that the energy difference between both conformations is just −0.55 kcal mol^−1^, changes in dipole moments through reorientations through 7-OH positions may be easily produced at very low energy cost. Therefore, to analyze the rule of dipolar forces on the flavonoid/biomolecule interactions, the exact configuration rising from the geometry and characteristics of the interaction site should be analyzed [22]. Nevertheless, these molecules may lead to remarkable dipole – dipole interactions with the biomolecules’ specific sites through changes in their conformations leading to remarkable increases in their dipole moments.

### 3.2. Intramolecular Hydrogen Bonding. AIM and NBO Analysis

**Figure 4.**
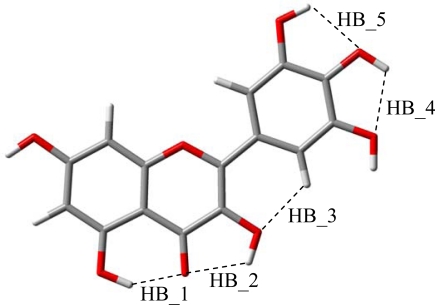
Sites for intramolecular hydrogen bonding in the studied compounds.

**Figure 5.**
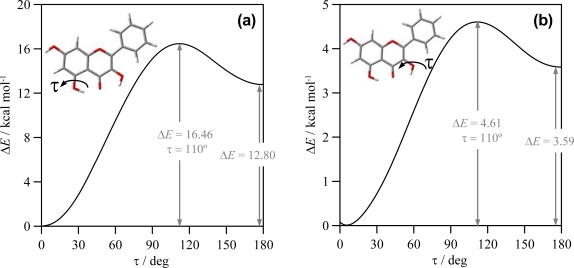
Relaxed potential energy scan for the reported dihedrals (involved in HB_1 and HB_2 as in Figure 4) in flavonoid IX (galangin), calculated in gas phase at B3LYP//6-311++G** theoretical level. Δ*E* is the energy relative to the conformer with lower energy (τ = □0°).

The reported results for the studied flavonoids show that up to five intramolecular hydrogen bonds are possible, HB_1 to HB_5, Figure 4. In this section, the characteristics of these hydrogen bonds will be analyzed. The strength of these bonds may be quantified through relaxed potential energy scans for hydroxyl groups involved in each interaction. In Figure 5, these torsional profiles are reported for HB_1 and HB_2 in molecule IX (galangin).

**Figure 6.**
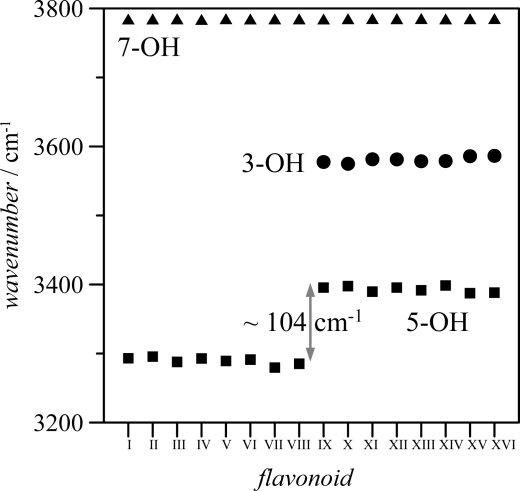
Frequencies of stretching vibrations of hydroxyl groups in 3, 5 and 7 positions (atom numbering as in Figure 1) for the studied flavonoids, calculated in gas phase at B3LYP//6-311++G** theoretical level. Reported values show calculated unscaled frequencies.

The results reported in Figure 5a for HB_1 show that this H-bond is remarkably strong, the energy difference between conformers with and without HB_1 is 12.80 kcal mol^−1^ and the interconversion of both structures evolves through a 16.46 kcal mol^−1^ torsional barrier. Previously reported results at AM1 theoretical level [39] reported a destabilization energy of 6.04 kcal mol^−1^ for quercetin because of the absence of HB_1: as it has been reported in the previous section, HB_1 is almost unaffected by the presence of hydroxyl groups in cathecol ring, and thus the strength of HB_1 should be almost constant for the studied compounds. Therefore, AM1 results reported [39] remarkably underestimated the strength of HB_1, and, considering also the results reported in the previous section, AM1 literature results for these compounds should be taken with caution, and only high level computations should be considered as reliable. Torsional analysis of HB_2 is reported in Figure 5b; this bond is also strong, although weaker than HB_1, and the interconversion of conformers with and without HB_2 evolves though a remarkable barrier. albeit lower than for HB_1. AM1 results in Ref. [39] reported a 3.65 kcal mol^−1^ energetic difference for the HB_2 in quercetin, which is in surprising agreement with the 3.58 kcal mol^−1^ reported in this work. The weaker character of HB_2 is in agreement with the shorter O-H interatomic distance for the OH group in position 3 than for the group in position 5, Table 2; the longer the OH interatomic distance, the stronger the hydrogen bonding. Moreover, the interaction of 3-OH with 6′-H, should lead to a weakening of 3-OH interaction with the keto group oxygen to allow the interaction with the 6′-H atom, at least for one of the lonely pairs of the 3-OH oxygen. Stretching vibrational frequencies of 3-OH and 5-OH groups also show remarkable differences between both groups, for molecule IX these frequencies appear at 3577. 5 and 3395.6 cm^−1^ (unscaled values), respectively, pointing to a stronger hydrogen bonding for 5-OH group. This behavior is confirmed by the calculated values of hydroxyl stretching vibrational frequencies reported in Figure 6 for 3-OH, 5-OH and 7-OH groups in the 16 studied flavonoids.

The frequency order is 7-OH > 3-OH > 5-OH. The almost constant values of 7-OH frequencies (∼3785 cm^−1^ for the 16 flavonoids) discard any hydrogen bonding through this position. The larger values of frequencies for 3-OH in comparison with 5-OH group point to a stronger hydrogen bonding between keto group oxygen and 5-OH for all the studied flavonoids; nevertheless, the introduction of an hydroxyl group in position 3 also weakens 5-OH interaction with keto groups, as it may be inferred from the blue-shifting of 5-OH vibrational frequencies for molecules IX–XVI in comparison with molecules I–VIII. Therefore, the main effect on hydrogen bonding in the studied molecules is the presence or absence of hydroxyl group in position 3, because the hydroxyl groups in B-ring do not affect remarkably the interactions in A and C rings. Stretching vibrations for the hydroxyl groups in B ring are of 3,750–3,800 cm^−1^ (unscaled values) for groups in 3′, 4′ and 5′, for all the studied compounds, and thus, in the same range as 7-OH vibrations, discarding strong hydrogen bonding for these hydroxyl groups.

**Figure 7.**
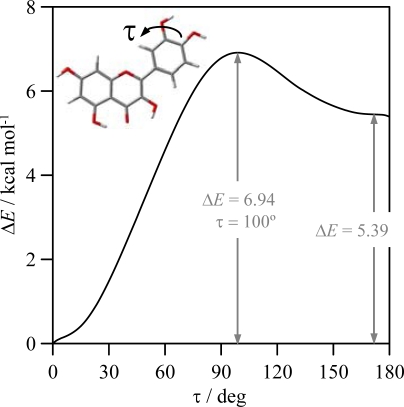
Relaxed potential energy scan for the reported dihedral (involved in HB_5 as in Figure 4) in flavonoid XIII (quercetin), calculated in gas phase at B3LYP/6-311++G** theoretical level. Δ*E* is the energy relative to the conformer with lower energy (τ = 0°).

The strength of HB_3 was analyzed in the previous section through the torsional profiles of (3-2-1’-6’) dihedrals, this hydrogen bonding has a strength of around 1 kcal mol^−1^, and thus, it is clearly weaker than HB_1 and HB_2. Finally, possible hydrogen bonding between OH groups in the catechol moiety (HB_4 and HB_5), are analyzed in Figure 7, in which results of a relaxed potential energy scan in quercetin is reported. Torsional profile reported in Figure 7 shows that interactions between neighbour hydroxyl groups in B-ring are clearly weaker than HB_1 but stronger than HB_2 and HB_3.

**Figure 8.**
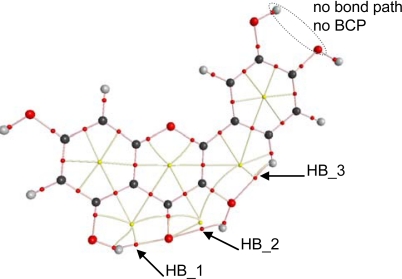
AIM analysis for flavonoid XIII (quercetin). Small red dots represent bond critical points, small yellow dots represent ring critical points, pink lines represent bond paths, yellow lines represent ring paths, and large dots represent attractors (atoms; red, oxygen; black, carbon and gray, hydrogen).

To get a deeper insight into the characteristics of intramolecular hydrogen bonding, AIM and NBO analysis were carried out for the optimized structures reported in Figure 2. The first evidence of hydrogen bonding according to the AIM approach is the existence of a bond path between two atoms and the existence of a bond critical point, BCP, in the middle of the path [44,45]. AIM results keep the Lewis bonding scheme for all the studied flavonoids, moreover, BCPs are found for HB_1, HB_2 and HB_3 but not for proposed HB_4 and HB_5, Table 4, as it is reported in Figure 8 for quercetin. These results are very surprising for HB_4 and HB_5 interactions, considering the behavior reported in Figure 7 pointing to a remarkable interaction through these positions, clearly stronger than those for HB_2 and HB_3 sites. These results for HB_4 and HB_5 interactions will be clarified in the next sections using both AIM and NBO results.

BCPs are not exactly placed in the middle of the bond paths for HB_1 to HB_3; instead they are shifted toward the hydrogen atoms involved in each hydrogen bond for all the studied flavonoids. A second AIM criterion to define and hydrogen bond considers that electron density at BCP, ρ_BCP_, and the Laplacian of electron density at BCP, ∇^2^ *ρ*_BCP_, must be within the 0.002–0.035 and 0.024–0.139 ranges, respectively (both in atomic units) [43,44]. This second criterion is fulfilled for HB_1, HB_2 and HB_3 in the 16 studied flavonoids, Table 4, and the ordering of these two properties is HB_1 > HB_2 > HB_3. Thus, this should be the strength ordering of these hydrogen bonds, which is in agreement with the results reported from torsional barriers. The main effect on ρ_BCP_, and ∇^2^ *ρ*_BCP_ values is the presence/absence of 3-OH group, and thus, for each hydrogen bond, these properties do not change from flavonoid I up to VIII (without 3-OH group) and from IX up to XVI (with 3-OH group), and are clearly different for these two groups of flavonoids. Moreover, ρ_BCP_, and ∇^2^ *ρ*_BCP_ for HB_1 decrease with the presence of 3-OH group, and thus, this hydrogen bond is weakened by the presence of this neighboring group. Diagonalization of the Hessian of the electron density yields three eigenvalues, λ_1_ < λ_2_ < λ_3_; the ellipticity, ɛ, is defined as λ_1_/λ_2_ − 1 and measures the extent to which charge is preferentially accumulated; these properties are reported in Table 5 for quercetin and luteolin, and similar results are obtained for the remaining studied flavonoids. Ellipticity is much larger for HB_2 than for HB_1, confirming that HB_2 is weaker, because large ɛ shows structural (topological) instability, and therefore a hydrogen bond that can easily be ruptured. The presence of a neighbor 3-OH group does not remarkably affect the ɛ value of HB_1, Table 5, in which results for luteolin (without 3-OH group) and quercetin (with 3-OH group) are reported. Ellipticity for HB_3 is low, even lower than for HB_1. Nevertheless, λ_1_ and λ_2_ values for HB_3 are around three times lower, in absolute value, than those for HB_1, Table 5, and thus, a lower concentration of charge density in HB_3, and therefore a weaker hydrogen bond, may be inferred. The larger values of λ_1_ and λ_2_ for HB_1 in flavonoids without 3-OH (luteolin in Table 5) point also to a lowering of charge density upon 3-OH group addition. λ_3_ parameter shows how easily the BCP can be moved along the bond path [45], and thus the larger this parameter, the stronger the hydrogen bond. Therefore, the lower values of λ_3_ also point to a weakening of HB_1 when 3-OH is added and to the weaker character of HB_3 in comparison with HB_1 and HB_2, Table 5. Another criterion for structural stability is the distance between a BCP and a ring critical point, RCP; RCP is close to BCP for HB_2, whereas the distance between BCP and the closer RCP is larger for HB_1 and HB_3, Figure 8, and thus, a greater instability for HB_2 may be inferred.

**Figure 9.**
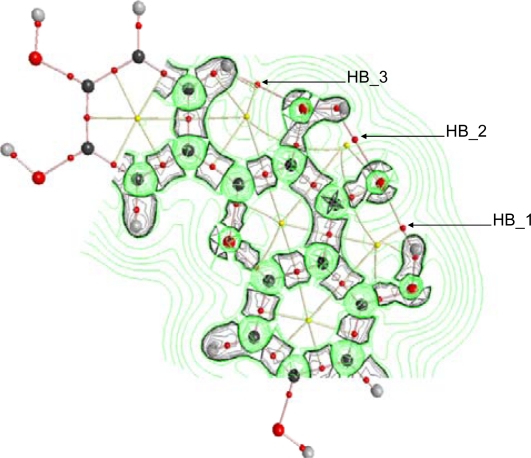
AIM analysis for flavonoid XIII (quercetin). Small red dots represent bond critical points, small yellow dots represent ring critical points, pink lines represent bond paths, yellow lines represent ring paths, large dots represent attractors (atoms; red, oxygen; black, carbon and gray, hydrogen). Contour plots represent laplacian of electron density, ∇^2^ *ρ*, in the vicinity of HB_1 to HB_3 hydrogen bonds; green curves, positive isosurface values of ∇^2^ *ρ* and black curves, negative isosurface values of ∇^2^ *ρ*. Contour plot in the vicinity of possible HB_5 is not represented because AIM analysis does not reveal any bond critical point in that region, and thus no hydrogen bond should be present according to this analysis.

The behavior of ∇^2^ *ρ* in the vicinity of HB_1, HB_2 and HB_3 is reported in Figure 9 for quercetin, although similar trends are obtained for flavonoids IX – XVI. ∇^2^ *ρ*is the sum of eigenvalues λ*_i_*, with ∇^2^ *ρ* < 0 values showing internuclear charge concentration corresponding to the so-called shared interactions (covalent bonds), whereas ∇^2^ *ρ* > 0 shows charge depletion corresponding to the so-called closed shell interactions (hydrogen bonding, ionic bonds and van der waals interactions). Valence shell charge concentration, VSCC, is not polarized for all the hydrogens involved in HB_1, HB_2 and HB_3, and thus, hydrogen bonds with purely electrostatic nature may be inferred. Contours plots of ∇^2^ *ρ* are slightly different in the region of HB_1 compared with HB_2, with larger charge depletion for HB_1, and thus pointing again to a stronger HB_1 hydrogen bond in comparison with HB_2.

The energetic properties of BCP associated with hydrogen bonding also provide valuable information about these interactions according to the AIM approach. Kinetic energy density, *G*, is always positive, and its ratio to electron density, *G/ρ_BCP_*, may be used to define the character of the interaction, being larger than 1.0 for closed shell and less than 1.0 for shared interactions [33]; this property is related to the ionic character of the interaction [46]. Potential energy density (or virial field), *V*, that is always negative, is related to the covalency of the interaction [46] and with hydrogen bonding strength through several empirical relationships [47,48]. It should be remarked the relationship between *V*, *G* and ∇^2^ *ρ* through the local form of the virial theorem [33]. Moreover, total energy density, *H*, the sum of *G* and *V*, should be positive for closed shell interactions, such as hydrogen bonding, indicating that kinetics energy density dominates the potential energy density, and negative for shared interactions [33]. *G* and *H* values are reported in Table 5 for quercetin and luteolin. The ratios *G/ρ_BCP_* are lower than 1.0 for HB_1, HB_2 and HB_3, although for closed shell interactions as these ones they should be larger than 1.0 [33]; nevertheless, values slightly lower than 1.0 have been previously reported for hydrogen bonding in other compounds [46]. *H* values are negative for HB_1, corresponding to strong hydrogen bonds, and slightly positive for HB_2 and HB_3. *H* for BCP in HB_1 decreases on going from luteolin to quercetin, and so *G* does, this is in agreement with the weakening of HB_1 upon addition of 3-OH group, the larger the *G* values, the stronger the hydrogen bonding. HB_2 and HB_3 show lower *G* values in comparison with HB_1, and thus they are weaker hydrogen bonds.

**Figure 10.**
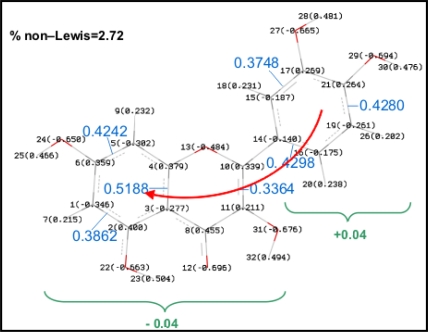
NBO analysis of charge delocalization in molecule XIII (quercetin). Parenthesized values for each atom indicate NBO charges, values in blue indicate occupancy of antibonding orbitals (BD*) for each bond, values in green indicate sum of NBO charges for each moiety (B ring and A + C), red arrow indicate mechanism of charge delocalization.

NBO results are reported in Table 6 and valuable information on the energy of studied hydrogen bonds may be inferred from them. Moreover, charge delocalization from A to B rings via C one (through the C2–C3 double bond) can be also analyzed using the NBO approach. Results reported in Figure 10 show a remarkably non-Lewis percentage for Quercetin, the population of antibonding orbitals, BD*, is also important and shows how the charge delocalization between A and B rings is done through the double bond in C ring. Analogous results are obtained for all the studied flavonoids, with a % non–Lewis character in the 2.6–2.9 range, and thus, an effective charge delocalization is obtained for all the studied molecules, including those slightly non–planar (I–VIII).

Hydrogen bonding may be analyzed considering a charge transfer between the donor and acceptor atoms. Therefore, hydrogen bonding arising from keto oxygen/hydroxyl hydrogen interactions, HB_1 and HB_2, and from hydroxyl/hydroxyl interactions, HB_4 and HB_5, should be determined by the hyperconjugation induced charge transfer between the corresponding oxygen electron lonely pairs (donor) and the antibonding orbitals for the hydroxyl bond (acceptor), *n_O_*→σ*_O–H_. For HB_3, hyperconjugation will be carried out between lonely pairs in hydroxyl oxygen and orbitals for hydrogen atom in position 6′. Three main properties are considered to analyze hydrogen bonding in the studied molecules according to the NBO method: *i*) second order perturbation energy, *E*(2), *ii*) energy difference among the donor and the acceptor, *ΔE*, and *iii*) Fock matrix element between the donor and acceptor, *F_ij_*. In agreement with the results reported in previous sections using different approaches, the main effect on NBO parameters for the studied hydrogen bonds is the absence/presence of a 3-OH group.

Properties of HB_1 do not change remarkably on going from flavonoids I to VIII, without 3-OH group, and do change remarkably upon 3-OH addition, flavonoids IX to XVI, especially for the interaction between the second oxygen pair. Properties of HB_1 also do no change by the presence of OH groups in the B ring. Hyperconjugation in HB_1 rises from two different keto oxygen lone pairs, pairs 1 and 2; *E*(2) for pair 2 is remarkably larger than for pair 1 because of the lower energy difference and the orbital overlapping between donor and acceptor orbitals, which is reflected in the larger *F_ij_* value for pair 2. The large value of *E*(2) should be the molecular origin of the remarkable strength for HB_1. Nevertheless, HB_1 strength decreases upon 3-OH addition, the properties for interaction through pair 1 do not change when 3-OH group is added, but for pair 2 they change remarkably: the energy difference between donor and acceptor is the same both with and without 3-OH but the Fock matrix element decreases. Therefore, the molecular origin of the weakening of HB_1 upon 3-OH presence is the loss of orbital overlapping ability between donor and acceptor that hinders the hyperconjugation induced charge transfer between the second pair in keto oxygen and OH in position 5 antibonding orbitals. For HB_2, NBO results show that it is clearly weaker than HB_1, with stronger interaction through pair 2 but with *E*(2) values lower than for HB_1 for both pairs. The reason for this weaker character of HB_2 stands in the poorer orbital overlapping between donor and acceptor for both pairs, lower *F_ij_* values, in spite of the equal values of *ΔE* for HB_2 and HB_1. In Table 7, the main properties of molecular orbitals involved in HB_1 for flavonoid V (luteolin) and HB_1 and HB_2 for flavonoid XIII (quercetin) are reported. The main differences between orbitals corresponding to lonely pairs 1 and 2 (LP-1 and LP-2) in 4-*O* oxygen, for both flavonoids, are: *i*) LP-1 has a remarkably lower energy, which hinders hyperconjugation induced charge transfer with antibonding orbitals in 5-OH and 3-OH positions and *ii*) LP-1 has a mixed s/p character, whereas LP-2 is an almost purely p orbital. The *F_ij_* value is larger for transferences from LP-2, and thus the increasing p character of donor orbital seems to favour the efficiency of charge transference. The remarkably lower *F_ij_* value for the transference from LP-2 toward 3-OH orbital in comparison with that toward 5-OH, which leads to the lower strength of HB_2, can not be justified by the energies of acceptor OH orbital because the eigenvalues for 3-OH and 5-OH are almost equal, and thus only the very subtle differences in the s/p characters of the OH acceptor orbitals could justify the weaker character of HB_2. The weakening of HB_1 upon 3-OH addition may be justified comparing the changes in donor/acceptor orbital for flavonoids V and XIII reported in Table 7. The energy of LP-2 orbital does not change and the s character decreases upon 3-OH addition. The energy of 5-OH acceptor orbital does not change also with the presence of 3-OH group and only very subtle differences in the s/p character rises in quercetin in comparison with luteolin. Therefore, these subtle changes in the characteristics of the donor/acceptor orbitals involved in HB_1 upon 3-OH addition may justify the remarkable decrease of HB_1 with the presence of neighbor 3-OH group and HB_2.

HB_3 hydrogen bonding is produced only through the interaction of pair 1 in 3-OH oxygen with 6′-H, this interaction is weak because of the large energy difference and the poor orbital overlapping between the acceptor and donor orbitals. HB_4 and HB_5 were not considered as true H-bonds from the AIM viewpoint, because of the absence of BCPs, in spite of the remarkable large energy stabilization reported in Figure 7. Results reported in Table 6 using NBO method are in full agreement with those obtained with AIM, donor/acceptor interactions for these hydrogen bonds are carried out only through pair 1 in the corresponding hydroxyl oxygen, and these interactions are not very effective mainly because of the poor orbital overlap between donor/acceptor orbitals, as the very low *F_ij_* values show. Moreover, results using AIM and NBO approaches are in full agreement with geometrical parameters reported in Tables 4 and 6 together with vibrational frequencies reported in Figure 6 and along the text:

- The interatomic O-H distances are 5-OH (HB_1) > 3-OH (HB_2) > 3′-OH, 4′-OH, 5′-OH (HB_4 and HB_5), Table 4. Moreover, these distances for 3′-OH, 4′-OH, 5′-OH are almost equal to those for 7-OH, for which no hydrogen bonding is present.
- Vibrational frequencies of OH groups are 5-OH (HB_1) < 3-OH (HB_2) < 3′-OH, 4′-OH, 5′-OH (HB_4 and HB_5), Table 4. Moreover, these frequencies for 3′-OH, 4′-OH, 5′-OH are almost equal to those for 7-OH, for which no hydrogen bonding is present.
- The donor/acceptor distances are HB_1 < HB_2 < HB_4, HB_5, Table 4.
- The donor/acceptor angles are HB_1 > HB_2 > HB_4, HB_5, Table 4.
- NBO results, Table 6, shows *E*(2) and *F_ij_* values HB_1 > HB_2 > HB_4, HB_5.

Therefore, all the results shows that the strength of the interactions is HB_1 > HB_2 > HB4, HB5. Nevertheless, results reported in Figure 7 show a remarkable torsional barrier and energy stabilization for HB_4 and HB_5. This apparent contradictory results could be explained considering the values of the interaction angles reported in Table 4, that show the lower values for HB_4 and HB_5, and thus leading to a poor donor/acceptor overlapping (low *E*(2) and *F_ij_* values), the absence of BCPs, and interatomic distances and angles pointing to weak interactions.

## 4. Conclusions

A systematic computational study on the properties of flavonoids has been carried out to get a deeper insight into their molecular properties. The complex intramolecular hydrogen bonding features have been analyzed from different approaches. The main conclusions that can be extracted from this work may be summarized as:

- the molecular planarity of the studied molecules is forced by the presence of 3-OH group through the development of a hydrogen bonding with 6′-H atom in cathecol B-ring.
- up to five hydrogen bondings may rise in function of the hydroxyl groups present in the studied molecules.
- hydrogen bonding between 5-OH and 4-O keto groups, HB_1, is the stronger one, although its strength decreases upon 3-OH addition.
- interaction between 3-OH and 4-O group leads to HB_2, which is remarkably weaker than HB_1.
- the main differences between HB_1 and HB_2 rise from the characteristics of the involved orbitals in both interactions.
- hydrogen bonding between hydroxyl groups in A and C rings is not affected by the presence of hydroxyl groups in B-ring.
- hydrogen bonding between 3-OH and 6′-H atom is a weak interaction. Nevertheless, it leads to the molecular planarity and increases the torsional barrier improving the π-delocalization from the C ring toward the catechol B-ring.
- interactions between neighbor hydroxyl groups in B-ring should be considered with caution as hydrogen bonds according to AIM and NBO analyses.

## Figures and Tables

**Table 1.** Flavone derivatives, flavonoids, together with the numbering code used throughout this work. The first row of numbers indicates the molecular position, with numbering as in Figure 1.

**Name**	**Compound**	**3**	**5**	**7**	**3′**	**4′**	**5′**
**Flavone**	-	H	H	H	H	H	H
	I	H	OH	OH	H	H	H
	II	H	OH	OH	OH	H	H
**Apigenin**	III	H	OH	OH	H	OH	H
	IV	H	OH	OH	H	H	OH
**Luteolin**	V	H	OH	OH	OH	OH	H
	VI	H	OH	OH	OH	H	OH
	VII	H	OH	OH	H	OH	OH
	VIII	H	OH	OH	OH	OH	OH
**Galangin**	IX	OH	OH	OH	H	H	H
	X	OH	OH	OH	OH	H	H
**Kaempfero**	XI	OH	OH	OH	H	OH	H
	XII	OH	OH	OH	H	H	OH
**Quercetin**	XIII	OH	OH	OH	OH	OH	H
	XIV	OH	OH	OH	OH	H	OH
	XV	OH	OH	OH	H	OH	OH
**Myricetin**	XVI	OH	OH	OH	OH	OH	OH

**Table 2.** Main geometrical and energetic parameters of the studied flavonoids in gas phase calculated at B3LYP/6-311++G** theoretical level. Atom numbering as in Figure 1. Interatomic distances, *r*, in Å; dihedral angles, τ, in deg; energy, *E*, in a.u.; dipole moment, *μ*, in *D*.

**Comp.**	***r*(C4=O)**	***r*(3-OH)**	***r*(5-OH)**	***r*(7-OH)**	***r*(3′-OH)**	***r*(4′-OH)**	***r*(5′-OH)**	***τ*(1-2-1′-2′)**	***E***	***μ***
–	1.227	–	–	–	–	–	–	21.59	−728.241463	4.51
**I**	1.247	–	0.989	0.965	–	–	–	21.05	−878.741762	3.85
**II**	1.247	–	0.989	0.965	0.965	–	–	19.62	−953.983707	4.58
**III**	1.248	–	0.990	0.965	–	0.965	–	17.20	−953.985249	3.78
**IV**	1.247	–	0.990	0.965	–	–	0.965	22.37	−953.983761	2.52
**V**	1.248	–	0.990	0.965	0.967	0.964	–	18.23	−1029.228473	4.90
**VI**	1.247	–	0.990	0.965	0.965	–	0.965	21.14	−1029.226225	3.19
**VII**	1.248	–	0.990	0.965	–	0.968	0.964	16.02	−1029.228953	2.46
**VIII**	1.248	–	0.990	0.965	0.968	0.967	0.964	18.27	−1104.472305	3.63
**IX**	1.256	0.977	0.984	0.965	–	–	–	0.00	−954.014931	2.29
**X**	1.256	0.977	0.984	0.965	0.965	–	–	0.60	−1029.228052	2.45
**XI**	1.257	0.977	0.985	0.965	–	0.965	–	0.00	−1029.230044	1.66
**XII**	1.256	0.978	0.984	0.965	–	–	0.965	0.00	−1029.229193	1.67
**XIII**	1.257	0.977	0.985	0.965	0.967	0.964	–	0.00	−1104.473337	2.71
**XIV**	1.256	0.978	0.984	0.965	0.965	–	0.965	0.00	−1104.471657	1.06
**XV**	1.257	0.977	0.985	0.965	–	0.968	0.964	0.00	−1104.474897	0.18
**XVI**	1.257	0.978	0.985	0.965	0.967	0.967	0.964	0.00	−1179.718130	1.50

**Table 3.** Maxima of the torsional barrier, Δ*E*_max_, for the rotation of the (3–2–1′–6′) dihedral angle in the studied flavonoids computed in gas phase at the B3LYP/6-311++G** theoretical level. All the maxima appear at τ = 90° (Figure 3).

**Compound**	Δ*E*_max_**/kcal mol**^−^**^1^**	**Compound**	Δ*E*_max_**/kcal mol**^−^**^1^**
	
**Flavone**	3.44	IX	4.59
**I**	3.48	X	4.67
**II**	3.50	XI	5.09
**III**	3.83	XII	4.41
**IV**	3.39	XIII	5.01
**V**	3.77	XIV	4.59
**VI**	3.46	XV	4.64
**VII**	3.66	XVI	5.71
**VIII**	3.84		

**Table 4.** AIM analysis of studied flavonoids monomers computed in gas phase at the B3LYP/6-311++G** theoretical level. Electron density at BCP, *ρ*_BCP_; Laplacian of electron density at BCP, ∇^2^ *ρ*_BCP_; donor–acceptor interatomic distance, *r*, and angle, *ϕ*, for the intramolecular H-bonds. Values calculated for optimized structures reported in Figure 2. Intramolecular H-bonding sites defined in Figure 4. *ϕ* is defined ad D-H---A, where it stands for O-H---O(C) for HB_1 and HB_2, C-H---O(H) for HB_3, and O-H---H(O) for HB_4 and HB_5.

**Comp.**	**H-bond**	*ρ_BCP_***/a.u.**	∇^2^*ρ_BCP_***/a.u**	***r*/Å**	*ϕ***/deg**	**Comp.**	**H-bond**	*ρ_BCP_***/a.u.**	∇^2^*ρ_BCP_***/a.u**	***r*/Å**	*ϕ***/deg**
**I**	HB 1	0.044	0.128	1.74	146.9	XII	HB 1	0.038	0.119	1.80	145.7
**II**	HB 1	0.044	0.128	1.74	146.9		HB 2	0.026	0.103	2.02	117.7
**III**	HB 1	0.044	0.128	1.74	147.0		HB 3	0.018	0.076	2.13	122.6
**IV**	HB 1	0.044	0.128	1.74	146.8	XIII	HB 1	0.038	0.120	1.80	145.9
**V**	HB 1	0.044	0.128	1.74	147.0		HB 2	0.026	0.100	2.02	118.0
	HB 5	No	No	2.16	112.0		HB 3	0.018	0.076	2.15	123.3
**VI**	HB 1	0.044	0.128	1.74	146.8		HB 5	No	No	2.17	112.1
**VII**	HB 1	0.044	0.128	1.74	147.0	XIV	HB 1	0.038	0.120	1.80	145.6
	HB 4	No	No	2.15	112.3		HB 2	0.026	0.103	2.01	118.2
**VIII**	HB 1	0.044	0.128	1.74	147.0		HB 3	0.018	0.074	2.13	123.9
	HB 4	No	No	2.21	110.3	XV	HB 1	0.039	0.120	1.80	145.9
	HB 5	No	No	2.21	111.7		HB 2	0.026	0.104	2.02	118.1
**IX**	HB 1	0.038	0.119	1.80	145.8		HB 3	0.018	0.075	2.13	124.0
	HB 2	0.026	0.104	2.02	117.9		HB 4	No	No	2.15	112.4
	HB 3	0.018	0.076	2.15	123.8	XVI	HB 1	0.039	0.119	1.80	145.8
**X**	HB 1	0.038	0.120	1.80	145.7		HB 2	0.026	0.103	2.02	118.3
	HB 2	0.026	0.104	2.02	118.0		HB 3	0.018	0.074	2.13	123.7
	HB 3	0.018	0.072	2.16	123.4		HB 4	No	No	2.21	110.5
**XI**	HB 1	0.039	0.120	1.79	145.9		HB 5	No	No	2.21	111.9
	HB 2	0.026	0.100	2.03	117.8						
	HB 3	0.018	0.076	2.15	123.5						

**Table 5.** Eigenvalues, λ*_i_*, of hessian of electron density; ellipticity, ɛ; kinetic energy density, *G*; potential energy density (or virial field), *V*; total energy density, *H*; and ratio between *G* and electron density at BCP, *ρ*_BCP_; computed at the corresponding BCPs for each hydrogen bonding, according to the AIM analysis of flavonoid V (luteolin) and XIII (quercetin) in gas phase at the B3LYP/6-311++G** theoretical level.

**H-bond**	**λ_*1*_**	**λ_*2*_**	**λ_*3*_**	**ɛ**	***G***	***H***	***V***	***G/***ρ**_BCP_**
**HB 1**	−0.059	−0.058	0.236	0.014	0.0318	−0.002	−0.034	0.84

HB 1*	−0.072	−0.071	0.271	0.016	0.0363	−0.004	−0.041	0.83
**HB 2**	−0.031	−0.019	0.152	0.666	0.0233	0.002	−0.021	0.90
**HB 3**	−0.019	−0.019	0.111	0.008	0.0155	0.003	−0.013	0.86

* Results for luteolin.

**Table 6.** NBO analysis of studied flavonoids monomers computed in gas phase at the B3LYP/6-311++G** theoretical level. Second order perturbation energy, *E*(2); energy difference among the donor and the acceptor, Δ*E*; and Fock matrix element between the donor and the acceptor, *F_ij_*. Values calculated for optimized structures reported in Figure 2. Intramolecular H-bonding sites defined in Figure 4. The donor pairs belong to the oxygen and the acceptor is H–O (for HB_1, HB_2, HB_4 and HB_5) or H–C (for HB_3) for each H-bonding proposed in Figure 4. Reported values only for *E*(2) > 0.50 kcal mol^−1^.

**Comp.**	**H-bond**	**Donor pair**	***E*(2)/kcal mol^−1^**	Δ*E***/a.u.**	***F*_*ij*_/a.u.**	**Comp.**	**H-bond**	**Donor pair**	***E*(2)/kcal mol^−1^**	Δ*E***/a.u.**	***F*_*ij*_/a.u.**
**I**	HB 1	1	2.61	1.07	0.047	**XII**	HB 1	1	2.61	1.11	0.048
		2	18.25	0.68	0.101			2	13.10	0.68	0.086
**II**	HB 1	1	2.60	1.07	0.047		HB 2	1	0.60	1.10	0.022
		2	18.17	0.68	0.101			2	3.60	0.67	0.045
**III**	HB 1	1	2.63	1.07	0.048		HB 3	1	1.61	1.09	0.039
		2	18.45	0.68	0.102			2	No	No	No
**IV**	HB 1	1	2.62	1.07	0.047	**XIII**	HB 1	1	2.60	1.11	0.047
		2	18.35	0.68	0.101			2	13.35	0.68	0.087
**V**	HB 1	1	2.63	1.07	0.048		HB 2	1	0.60	1.10	0.023
		2	18.41	0.68	0.102			2	3.57	0.67	0.045
	HB 5	1	0.80	1.03	0.026		HB 3	1	1.62	1.09	0.038
		2	No	No	No			2	No	No	No
**VI**	HB 1	1	2.63	1.02	0.047		HB 5	1	0.79	1.03	0.025
		2	18.20	0.68	0.101			2	No	No	No
**VII**	HB 1	1	2.63	1.02	0.047	**XIV**	HB 1	1	2.62	1.11	0.049
		2	18.24	0.68	0.102			2	13.25	0.68	0.086
	HB 4	1	0.82	1.03	0.029		HB 2	1	0.60	1.10	0.023
		2	No	No	No			2	3.59	0.67	0.047
**VIII**	HB 1	1	2.63	1.02	0.048		HB 3	1	1.62	1.09	0.039
		2	18.25	0.68	0.103			2	No	No	No
	HB 4	1	0.81	1.03	0.027	**XV**	HB 1	1	2.61	1.11	0.049
		2	No	No	No			2	13.30	0.68	0.087
	HB 5	1	0.79	1.03	0.025		HB 2	1	0.61	1.10	0.023
		2	No	No	No			2	3.61	0.67	0.044
**IX**	HB 1	1	2.60	1.11	0.048		HB 3	1	1.62	1.09	0.040
		2	13.18	0.68	0.087			2	No	No	No
	HB 2	1	0.60	1.10	0.023		HB 4	1	0.82	1.03	0.031
		2	3.58	0.67	0.045			2	No	No	No
	HB 3	1	1.60	1.09	0.037	**XVI**	HB 1	1	2.60	1.11	0.048
		2	No	No	No			2	13.32	0.68	0.087
**X**	HB 1	1	2.59	1.11	0.048		HB 2	1	0.60	1.10	0.023
		2	13.07	0.68	0.086			2	3.58	0.67	0.046
	HB 2	1	0.60	1.10	0.023		HB 3	1	1.61	1.09	0.041
		2	3.62	0.67	0.045			2	No	No	No
	HB 3	1	1.58	1.09	0.037		HB 4	1	0.80	1.03	0.029
		2	No	No	No			2	No	No	No
**XI**	HB 1	1	2.62	1.11	0.048		HB 5	1	0.81	1.03	0.027
		2	13.40	0.68	0.087			2	No	No	No
	HB 2	1	0.58	1.10	0.022						
		2	3.50	0.67	0.045						
	HB 3	1	1.62	1.09	0.038						
		2	No	No	No						

**Table 7.** Characteristics of orbitals involved in hydrogen bonding HB_1 and HB_2 for flavonoids V (luteolin) and XIII (quercetin) computed in gas phase at the B3LYP/6-311++G** theoretical level. LP = oxygen lonely pair. Atom numbering as in Figure 1.

**orbital**	**eigenvalues**	**s/p character (%)**
**flavonoid XIII**		

4-O/LP-1	−0.7204	61.0/40.0
4-O/LP-2	−0.2971	0.6/99.4
5-OH/σ* (23.1% O + 76.8% H)	0.3856	O, 23.9/76.1
		H, 100.0/0.0
3-OH/σ* (24.4% O + 75.6% H)	0.3762	O, 21.4/78.6
		H, 100.0/0.0

**flavonoid V**		

4-O/LP-1	−0.6788	57.7/42.3
4-O/LP-2	−0.2926	2.8/97.2
5-OH/σ* (22.7% O + 77.3% H)	0.3875	O, 24.1/75.8
		H, 100.0/0.0
